# Technological Solutions for Diagnosis, Management and Treatment of Alzheimer’s Disease-Related Symptoms: A Structured Review of the Recent Scientific Literature

**DOI:** 10.3390/ijerph19053122

**Published:** 2022-03-07

**Authors:** Davide Maria Cammisuli, Gabriele Cipriani, Gianluca Castelnuovo

**Affiliations:** 1 Department of Psychology, Catholic University, 20123 Milan, Italy; davide.cammisuli1@unicatt.it; 2 Neurology Division, Versilia Hospital, 55049 Lido di Camaiore, Italy; cprgrl@gmail.com; 3 Clinical Psychology Research Laboratory, Istituto Auxologico Italiano IRCCS, 28824 Verbania, Italy

**Keywords:** Alzheimer’s disease, mental health technology, digital devices, applications, sensors, virtual reality, empowerment

## Abstract

In people with Alzheimer’s disease (PwAD), there is a need for specific tools for the timely diagnosis, management, and treatment of symptoms. New technological solutions, including digital devices, application programs (apps), sensors and virtual reality, represent promising possibilities for objective and reliable assessment, monitoring and intervention strategies in this field. Our structured review presents an up-to-date summary of the technological solutions for the (i) diagnosis, (ii) management and (iii) treatment of AD-related symptoms. To this end, we searched electronic databases (i.e., PubMed, Web of Science, and Cochrane Library) for studies published over the last 10 years. Two authors of the review extracted data of interest. A total of eight manuscripts were included. In the last decade, a series of technological solutions across AD stages have been proposed. These include: (i) innovative strategies for the early detection of deficits in finger dexterity, visuo-spatial abilities (including spatial navigation), divided attention and instrumental autonomy; (ii) tools to activate the patient’s responsiveness in terms of alertness and mood improvement; and (iii) useful interventions for retrieving memories, increasing body movements and improving spatial cognition. Methodological limitations, mainly pertaining to the paucity of randomized controlled trials and comprehensive assessments, were observed. Advances in technology currently provide the potential for designing innovative methods for evaluating, controlling and handling AD-related symptoms. The co-creation of technological solutions with all stakeholders represents the best way to design effective strategies for PwAD.

## 1. Introduction

Dementia is a neurological condition affecting approximately 50 million people worldwide [1]. Because of the aging population, it currently represents a social and health emergency. Dementia is usually characterized by loss of cognitive function and decreased behavioral abilities to such an extent that it interferes with a person’s activities in daily living. Moreover, behavioral and psychological symptoms of dementia (BPSD) afflict the vast majority of patients, especially those with Alzheimer’s disease (AD) [2]. AD represents 60–80% of all dementia types [3] and is the sixth leading cause of death in the United States [4]. The presence of extracellular senile plaques of insoluble β-amyloid peptide (Aβ) and neurofibrillary tangles composed of phosphorylated tau protein (P-tau) in neuronal cytoplasm constitute the hallmarks of this neurological condition [5]. Pathogenesis is characterized by an insidious onset; degeneration tends to develop slowly and gradually and worsens over many years. Over 90% of people with AD (PwAD) manifest BPSD in mild stages of the disease, including depression, anxiety, apathy, agitation and aggression; disinhibition, delusion, hallucinations, irritability and emotional lability; euphoria; and aberrant motor, sleep and eating behaviors [2,6].

Symptoms depend on the progression of the disease, which is divided into five stages: preclinical; mild cognitive impairment (MCI) *due to* AD; and mild, moderate and severe dementia *due to* AD. Preclinical AD is usually identified only in clinical settings thanks to the use of neuroimaging techniques able to detect amyloid-beta deposits without overt cognitive deficits. Conversely, individuals with MCI usually show low performance in objective neuropsychological testing of episodic memory, with difficulties that may also encompass other cognitive domains (e.g., executive functioning, visuo-constructive abilities, gnosis, praxis and language), without the impairment of personal and instrumental autonomy. In the mild stage, memory deterioration and difficulties in problem solving, complex tasks and judgment (e.g., making financial decisions) become more pronounced, and changes in personality (uncharacteristic irritability or anger, reduced motivation, etc.), difficulty in organizing and in expressing thoughts and getting lost or misplacing belongings may occur. During the moderate stage, PwAD display confusion and need more assistance in their daily activities. They especially show substantial difficulties in temporal orientation and a reduction in cognitive efficiency, require assistance for self-care and undergo significant changes in personality and behavior (e.g., wandering). Finally, at the severe stage, PwAD lose the ability to communicate coherently and require total assistance for eating, dressing, using the bathroom and performing self-care tasks. They may experience a deficit in motor abilities pertaining to walking and lose the ability to swallow and control bladder and bowel functions [7,8]. 

Physicians of PwAD thus have to deal with a complex spectrum of patient symptoms during the course of the disease, and taking care of demented patients is difficult and often results in depression, burden and compromised health for caregivers because of continuous and stressful daily care and support [6]. 

AD can be especially challenging to detect at an early stage. In addition to physical and neurological examination and a review of medical history, assessment includes neuropsychological tests, laboratory exams and neuroimaging investigations, resulting in a multidimensional and time-consuming practice including different clinical competences. To this end, researchers are working on new diagnostic tools that may enable clinicians to accurately diagnose AD earlier in the course of the disease when symptoms are very mild or even before symptoms appear [9], with positive implications for therapeutic purposes. 

Caregiver burden is associated with the higher dependence level of the patient and loss of autonomy, as well as BPDS severity [10,11]. Due to cognitive and progressive behavioral deterioration, a number of safety issues have been reported in the literature regarding PwAD, including injuries (e.g., falls, ingestion of dangerous substances, accidents with sharp objects, fires and burns) requiring hospital admission to the emergency department, abnormal behavior and misuse of medication [12]. Moreover, language skills as well as interactive communication decrease during AD progression, making patients unable to relate their needs to caregivers effectively [7,8]. To this end, beyond diagnosis, increasing attention has also been given to developing technologies for management (e.g., safety and security, mobility aids, support in everyday living tasks and engagement in familial and social relationships) and treatment (e.g., medication reminders and improvement in cognitive function and physical mobility) [13] of PwAD.

Technology can be used to empower PwAD, allowing them to live a more satisfying and meaningful life. People should be empowered to promote their own health, interact effectively with health services and be active partners in making decisions. Empowerment is a multidimensional community process through which individuals and groups are able to better understand, control and manage symptoms throughout their lives. It represents a key concept in the World Health Organization’s vision of health promotion. WHO consensus documents [14,15] on mental health identify the empowerment of people with mental difficulties (including cognitive ones) and those who care for them as key priorities in the health agenda for the next decades. Research should provide a deep understanding for mental health practitioners, decision-makers and urban service organizations about how PwAD and their caregivers can benefit from new technology products in order to improve cognitive health.

Our structured review aims to present an up-to-date overview of technological solutions in terms of digital devices, applications (apps), sensors and virtual reality (VR) options by highlighting their applicability for the diagnosis, management and treatment of AD-related symptoms. Implications for the empowerment of patients and their caregivers by designing effective strategies for AD care are discussed. 

## 2. Materials and Methods

**Figure 1:**
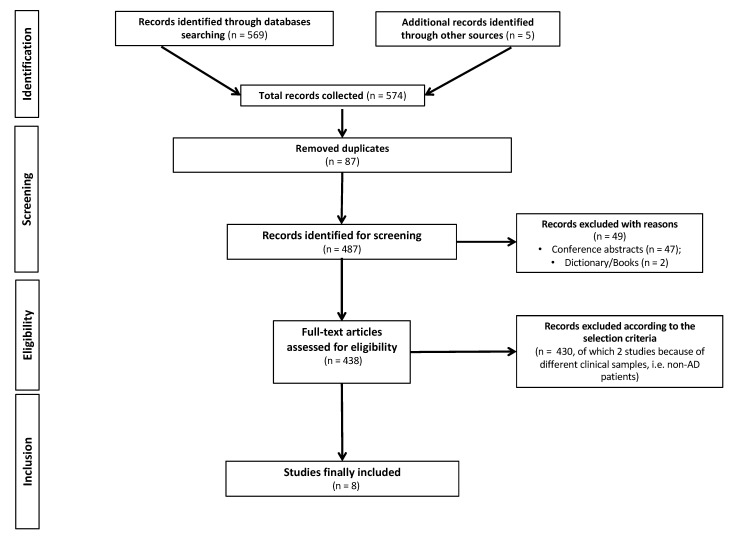
PRISMA (Preferred Reporting of Systematic Reviews and Meta-analysis) flowchart of search strategy and results.

The literature reviewed in this research was obtained by searching PubMed, Cochrane Library and Web of Science. A search strategy limited to the last 10 years (from 1 January 2012 to 19 January 2022) was performed using the following combination of terms: “Alzheimer’s Disease” AND “mental health technology” AND “digital devices” OR “app” OR “sensors” OR “virtual reality” AND “empowerment”. The search was restricted to a limited time period (i.e., 10 years) because technological solutions—including those designed for PwAD—have quickly improved over time. A total of 574 manuscripts were selected. Studies were eligible for inclusion if they reported effective technological solutions in terms of digital devices, apps, sensors or virtual reality (VR) for the diagnosis, management and treatment of AD-related symptoms. Exclusionary criteria encompassed the following: (i) non-primary research (i.e., scoping reviews, systematic reviews, meta-analyses, perspectives or case studies and research protocols); (ii) studies that analyzed results by qualitative methodologies; (iii) studies that included combined interventions (e.g., technological solutions plus pharmacological/non-pharmacological interventions); (iv) studies that included patients other than AD (i.e., other dementia types) or generally referred to subjects as ‘‘demented patients’’; and (v) studies written in languages other than English. This screening finally yielded 8 articles for evaluation (Figure 1). Two independent reviewers (D.M.C. and G.C.) extracted data of interest, and disagreements were discussed until a consensus was reached. 

## 3. Results

Over the last 10 years, advances in technology have led to innovative solutions for the diagnosis [16,17,18,19,20], management [21] and treatment [22,23] of PwAD. A summary of the main findings is shown in Table 1.

The results generally provide support for the applicability of technological solutions for the early detection of deficits in finger dexterity, visuo-spatial abilities (including spatial navigation), divided attention and instrumental autonomy for managing the patient’s behavior (i.e., alertness and mood improvement) and treatment interventions for retrieving memories and increasing body movements, as well as improving spatial cognition. The applicability of technological solutions covers all AD stages, i.e., preclinical [18,19], mild-to-moderate [16,17,20,22] and moderate-to-severe [21,22], with different implications for clinicians and caregivers.

The analysis of the selected studies involved some methodological limitations. Diagnostic criteria differed among studies, and they were not reported in two cases [21,22]. Two studies [16,18] based the AD diagnosis on the National Institute on Aging/Alzheimer’s Association (NIA-AA) criteria [24], and three studies [17,19,23] based it on the National Institute of Neurological and Communicative Disorders and Stroke and the Alzheimer’s Disease Related Disorders Association (NINCDS-ADRDA) criteria [25], with the supplement of the Diagnostic and Statistical Manual of Mental Disorders Fourth Edition (DSM-IV) [26] criteria in one case [20]. Heterogeneity among participants was also found. Enrolled individuals were outpatients in two cases [16,18]; in other cases, they were recruited from memory clinics [17], Alzheimer’s day centers [20,22] or social senior centers [19,23]. A convenience sample was used in another case [21]. Randomized controlled trials were not the rule, except for one investigation [23]; two studies compared experimental and control groups [16,19]. Three studies [17,18,20] aimed to reveal significant differences across the continuum of physiological/pathological aging among groups of healthy older adults, MCI and AD patients, while two studies drew conclusions from a single sample test [21,22]. In one study, the sample size was very small [22]. A comprehensive neuropsychological assessment was performed only in three studies [16,19,23], and no other neurophysiological or neuroimaging evaluation was adopted by researchers to corroborate their findings for the other selected studies. Pharmacological treatment able to influence patients’ performance was described only in one study [16], and recruited participants were generally poorly defined with regard to possible comorbidities they may have. Finally, compliance with technology was generally analyzed by using qualitative data (e.g., participants’ perception towards technology).

## 4. Discussion

Today, technological solutions represent useful tools to improve the diagnostic process, management and treatment of AD-related symptoms. Constant advances in technology provide the potential for designing innovative strategies for the diagnosis of AD-related symptoms. Intelligent and smart approaches for the diagnosis of AD are welcome, and technological innovations can be fast, low-cost and easy-to-administer solutions. Despite some methodological limitations of the selected studies, our review revealed positive findings in terms of technology applications to manage AD-related symptoms.

Given that not only higher brain functions but also fine motor skills are affected in AD [27], a finger-to-thumb tapping task represents a useful method for evaluating finger dexterity, which is believed to influence self-care and IADLs and may decline as AD progresses. Moreover, recent studies have shown that deterioration in performing IADLs may be an early predictor of cognitive deterioration and play a critical role in the progression of MCI to AD [28]. Intelligent and smart approaches for the diagnosis of IADLs are also represented by 3D cameras for video data analysis that are fast, low-cost and easy to install in different settings, including homes, with positive implications for the ecological validity of behavioral assessments. Our review also revealed that robotic interfaces and wearable sensors are valid tools for evaluating visuo-motor abilities and divided attention, respectively, offering more support for the neuropsychological assessment of PwAD.

Moreover, VR is becoming an established tool in AD clinical research, too [29]. Thanks to the ability of computer simulations to replace the external sensory world with an artificial environment or replicate the body movements of the user in an immersive way, the intuitive nature of this technology makes it appealing even to those with a significant burden of disease, such as PwAD. It is also useful both for the diagnosis and treatment of spatial navigation deficits that may occur in early stages [30]. Technical equipment integrating a computer system with a screen and sound amplifier, a pressure microswitch, a voice-detecting sensor with a throat microphone and basic software has also been shown to be consistent with patients’ behavior in the absence of engagement, producing encouraging results in fostering verbal abilities and arm raising in PwAD and offering new perspectives on the treatment of advanced stages of the disease.

AD is a chronic condition that causes the affected person to enter a state of progressive deterioration and become more dependent on others in order to continue performing personal and instrumental activities of daily living at a certain level. The usage of technology, such as tablet applications for the management of AD-related symptoms involving music therapy, may facilitate care planning and increase the empowerment of patients and their caregivers. It has been suggested that such an application allows caregivers to have a deep understanding of patients’ life experiences and may help them in maintaining their own health [31]. Empowerment in AD care can be defined as a confidence-building process whereby PwAD are respected, have a voice and are heard, are involved in making decisions about their lives and have the opportunity to create change through access to appropriate resources [32]. The process of designing technological solutions should be initiated as early as possible in collaboration with specialists as a comprehensive medical examination, including aspects that are important to PwAD, in order to clarify individual values, wishes and special needs for care.

## 5. Conclusions

According to the best practice guidelines for the involvement of people with dementia in developing technology-based solutions [33], a rigorous field-testing phase followed by observations by an expert team, exploration of the main methods for collecting feedback from PwAD and offering participants the opportunity to learn a new skill through their commitment may enhance patients’ wellbeing and improve empowerment. The co-creation of technological solutions in research by using brainstorming, focus groups, questionnaires, detailed interviews and surveys with all relevant stakeholders, including physicians, informal caregivers, staff members and technology developers, make the evaluation of the impact of the technology more effective and ensure that the system meets a high standard of sustainability and reliability in the diagnosis, management and treatment of PwAD. Having access to planning, information and resources and ensuring high-quality care represent key features of user empowerment in cognitive health, including in the case of PwAD.

## Figures and Tables

**Table 1:** Summary of the main findings of selected studies.

Type of Application	Reference and Study Purpose	Clinical Sample	Technical Specifications of the Tool	Main Findings	Implications for Clinicians or Caregivers
Omni^®^, Sensable (SIRS Lab, Siena)	Bartoli et al., 2017 [16] Diagnosis (assessment of visuo-motor deficits)	20 AD outpatients (mean age 74.2 ± 6.3 years) and 20 healthy age-matched controls	A low-cost robotic interface that can measure reaction times, tracking errors and proprioceptive deficits without a need for other recording devices	Movement planning requiring visuo-spatial recalibration is already compromised in AD	The Omni robot is a low-cost force feedback device that can be placed on any desktop and easily used in ambulatory clinics or at bed side for patients with mild AD
iTMT platform	Zhou et al., 2017 [17] Diagnosis (assessment of divided attention in a dual-task paradigm)	9 AD patients (mean age 80.8 ± 6.6), 10 MCI and 11 healthy older adults	An iTMT platform, inspired by the conventional paper-and-pencil Trail Making Test (Reitan, 1958). The platform has one wearable sensor (part of the LEGSys TM system, BioSensics, MA, USA), which includes a triaxial accelerometer, a gyroscope and a magnetometer for the estimation of angles and position. The sensor is attached to the subject’s shin. The use of an elastic strap allows tracking of ankle motion in 3D and translates it to a human–machine interface installed on a computer. By moving the ankle, the subject can navigate a cursor on the screen.	Feasibility and proof of concept of a simple, safe and practical iTMT system with promising results in identifying dual-task ability impairment among older adults with AD	The test is simple, short, safe and easy to administer, making it suitable for busy clinics
Just Touch; (Hitachi Maxell, Tokyo, Japan)	Suzumura et al., 2018 [18] Diagnosis (assessment of finger dexterity)	31 AD outpatients (mean age 74.2 ± 6.3 years), 15 MCI and 48 healthy older adults	A tablet application for detecting abnormalities of finger dexterity aimed at detecting (sounded) rhythmic tapping (one hand, both hands, simultaneously or alternating)	Decline in finger dexterity can reflect declining cognitive functioning	Finger dexterity parameters are associated with early cognitive decline
NeuroVirtual 3D	Serino et al., 2018 [19] Diagnosis (evaluation of spatial cognitive abilities related to executive attention system)	52 AD patients (mean age 84.4 ± 4.6) and 48 healthy older adults	NeuroVirtual 3D software (http://www.neruovirtual.eu, accessed on 20 January 2022) provides a free virtual-reality platform for easily customizing virtual environments from a predefined library of existing ones (park, supermarket, station, etc.). It is composed of two modules: the Editor, for the customization of virtual scenes, and the Player, for the visualization of customized scenes in immersive and non-immersive modalities.	The cognitive profile of AD appears to be characterized by an early decline in allocentric retrieval, combined with an early decline in other subtle neurocognitive mechanisms needed to support allocentric-to-egocentric switching, i.e., mental frame syncing, linked to brain changes occurring in hippocampal region and in retrosplenial cortex	This technology may enable early detection of cognitive impairments among individuals in the first stage of AD
Complex activity recognition (CAR) system by a 3D camera (ASUS Xtion Pro Live)	Karakostas et al., 2020 [20] Diagnosis (assessment of IADLs)	27 AD patients (mean age 73.8 ± 6.8 years), 38 MCI and 33 healthy older adults	Measure of IADL by a clinical protocol involving the following activities: prepare a drink; make a phone call to a specific number; establish account balance and transfer money through a tablet device to a specific account; prepare drug box following a prescription	Healthy controls significantly outperformed the MCI group, which had better performance compared to the AD group	The video data analysis can be used to assess IADL task quality and provide clinicians with objective measurements of patients’ performance
Tablet (Acer One-10 device, fitted with the Windows 10 operating system)	Lancioni et al., 2019 [21] Management (improving social engagement in advanced stages of AD for people who tend to be passive/detached and depressed) (sessions lasted 5 min, 3–5 times a day)	20 participants (mean age 82 years) recruited for the study	The tablet was supplied with basic, specifically arranged control software. Multiple music stimuli (preferred songs) were stored in the tablet memory so that the tablet could present them to the participants during sessions.	Participants’ hand responses were promoted, which enabled them to independently access preferred music and significantly increase social engagement	This technology can help formal and informal caregivers to more easily and extensively interact with patients with moderate-severe AD thanks to the amelioration of alertness and mood
Study 1 A technical apparatus consisting of a computer system with screen and sound amplifier, a pressure microswitch, a voice-detecting sensor with throat microphone and basic software Study 2 A technical apparatus consisting of a microswitch, a computer with sound amplifier and basic software	Lancioni et al., 2016 [22] Treatment (promoting positive verbal reminiscence) (sessions lasted 5 min, 2–4 times a day) Lancioni et al., 2016 [22] Treatment (promoting mild physical exercise) (sessions lasted 5 min, 2–4 times a day)	8 participants (mean age 82 years) recruited for the study 8 participants (mean age 82 years) recruited for the study	The participant sat in front of the computer screen, which showed photos or video clips of relevant people (including him- or herself) and special places and/or community and family events (e.g., wedding celebrations). The computer provided a brief verbal description of the photos and the videos and asked the participant to talk about them. A reminder to press the push button and talk more occurred after 10–20 s of the participant’s silence/passivity. Failure to activate the push button led the computer to provide additional reminders.The microswitch consisted of a tilt device or a combination of two such devices fixed to the participant’s arms. The computer’s use during baseline was limited to recording arm-raising responses. During the intervention, the computer: (a) delivered a 10 s stimulation after each arm-raising response; (b) presented a verbal reminder to raise the arms after no response after 15 s from the start of the session or from the end of a stimulation period; and (c) recorded arm-raising responses and reminders.	Significant improvement of verbal engagement after the intervention Significant improvement of arm-raising response after the intervention	Technology-aided programs can be used for supporting independent (i.e., computer-mediated) verbal engagement/reminiscence in mild-to-moderate AD. Technology-aided programs can be used for supporting mild physical exercise in mild-to-moderate AD.
NeuroVirtual 3D	Serino et al., 2017 [23] Treatment (a VR-based training program for the enhancement of spatial cognition) (10 session for 3–4 consecutive weeks)	20 AD patients (mean age > 65 years old) diagnosed as with (randomly assigned to an experimental and to a control group, 10 patients each), plus 8 healthy older adults	The software is composed of two main modules: the Editor, which permits the customization of pre-designed virtual environments (a city, an apartment, a supermarket, etc.) tailored to the specific needs of an experimental setting, and the Player, which allows the administration of the configured virtual environments	A significant improvement in long-term spatial memory after the VR-based training	Thanks to the Editor, researchers can customize virtual environments by choosing the appropriate stimuli from a database of objects (both 2D and 3D objects, videos and sounds)

AD = Alzheimer’s disease; iTMT = Instrumented Trail Making Test; MCI = mild cognitive impairment; IADL = instrumental activities of daily living.

## Data Availability

Not applicable.
